# Assessing biophysical and socio-economic impacts of climate change on regional avian biodiversity

**DOI:** 10.1038/s41598-021-82474-z

**Published:** 2021-02-08

**Authors:** Simon Kapitza, Pham Van Ha, Tom Kompas, Nick Golding, Natasha C. R. Cadenhead, Payal Bal, Brendan A. Wintle

**Affiliations:** 1 Quantitative and Applied Ecology Group, School of BioSciences, The University of Melbourne, Parkville, VIC 3010 Australia; 2 Crawford School of Public Policy, Australian National University, Acton, ACT 2601 Australia; 3 Centre of Excellence for Biosecurity Risk Analysis, School of BioSciences, The University of Melbourne, Parkville, VIC 3010 Australia; 4 NESP Threatened Species Recovery Hub, University of Melbourne and University of Queensland, St Lucia, QLD 4072 Australia

**Keywords:** Biodiversity, Biogeography, Climate-change ecology, Ecological modelling, Macroecology

## Abstract

Climate change threatens biodiversity directly by influencing biophysical variables that drive species’ geographic distributions and indirectly through socio-economic changes that influence land use patterns, driven by global consumption, production and climate. To date, no detailed analyses have been produced that assess the relative importance of, or interaction between, these direct and indirect climate change impacts on biodiversity at large scales. Here, we apply a new integrated modelling framework to quantify the relative influence of biophysical and socio-economically mediated impacts on avian species in Vietnam and Australia and we find that socio-economically mediated impacts on suitable ranges are largely outweighed by biophysical impacts. However, by translating economic futures and shocks into spatially explicit predictions of biodiversity change, we now have the power to analyse in a consistent way outcomes for nature and people of any change to policy, regulation, trading conditions or consumption trend at any scale from sub-national to global.

## Introduction

Climate change affects biodiversity through a multitude of pathways. There is pervasive evidence that climate change directly affects environmental conditions that are related to the climatic niches of many taxa, with the potential of significant shifts in their distributional ranges or even the total extinction of species^1,2^. However, climate change also affects biodiversity through indirect human-mediated impacts: it drives the loss of livelihoods and displacement^3^ and affects food and commodity production systems through its impacts on land productivity and human health^4,5^ and environmental suitability for different land uses^6,7^. Resulting global transitions of land use patterns are set to drive habitat conversion and may have dramatic impacts on biodiversity^8–10^. While there are some examples of studies examining synergistic effects of land use and climate change on species ^11,12^, large-scale assessments of biodiversity change in response to climate change have tended to look only at direct impacts of climate change on biophysical conditions or habitat loss and fragmentation alone^8^. Analyses that couple direct biophysical impacts on species with indirect socio-economic impacts via consumption, commodity, and land use change are sorely needed to fill important gaps in our knowledge of interactions between land use and climate change^10^, to foster a more holistic understanding of the impacts of climate change, and to support the design of cross-sectoral adaptation and mitigation strategies^13^.

No single model of drivers of change in biodiversity and ecosystem services can capture all relevant dynamics at a high level of detail and there is an increasing awareness of the urgency to consider interactions between direct and indirect drivers of change under future scenarios to characterise prospects and management options for biodiversity and ecosystem services^13^. Coupling demographic, economic and biophysical models has the potential to advance understanding and improve representation of synergies between direct and indirect drivers in biodiversity modelling, and to discover non-linear system behaviours that may not be apparent when considering drivers in isolation^13^.

**Figure 1:**
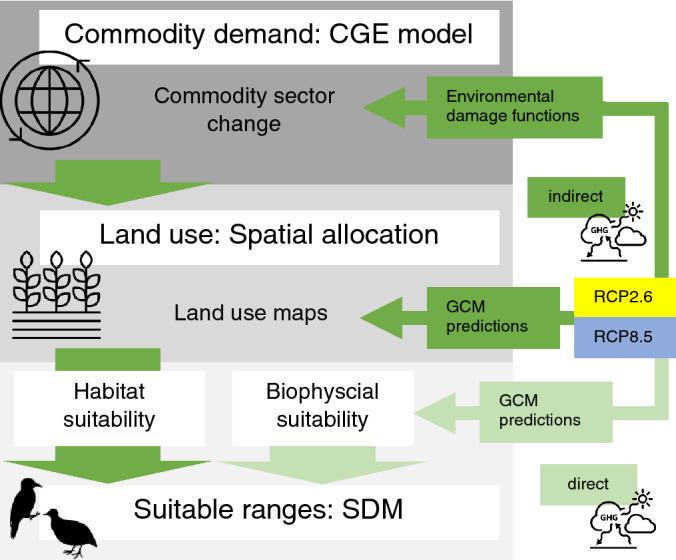
Overview of the modelling framework to capture interactions between direct and indirect drivers of biodiversity change under climate change scenarios. We included two Representative Concentration Pathways RCP2.6 and RCP8.5 to characterize the plausible extremes of climate change. Dark green arrows represent the indirect pathway of climate change impacts on suitable ranges. Light green arrows indicate the direct pathway of climate change impacts on ecological suitability. Icons from thenounproject.com.

Here, we contribute to the recent advances in integrated assessment modelling^14–17^ by applying an integrated modelling framework to compare the relative influence of direct biophysical and indirect socio-economic climate change impacts on the distribution and extent of suitable ranges for avian species in Vietnam and Australia (Fig. 1).

Recent advances in computable general equilibrium (CGE) modelling^18,19^ bring unprecedented power to parametrise the impacts of climate change on commodity consumption and production patterns at very high commodity and temporal resolution across the global economy. We combine this economic modelling power with state-of-the-art land use change modelling to spatially downscale commodity demand changes caused by climate change^4^ into changes of land use patterns. In order to isolate net climate change impacts on the economy, we parametrize only climate change damages in the CGE model, keeping all other economic parameters constant at current baseline values. The spatial realisation of changing land use patterns varies with changes in the suitability of land for particular uses and is thereby also driven by climate change^7,20^. Commodity demand changes are projected annually and land use predicted in 10-year time steps, producing decadal time-series maps of land use. Maps are integrated with climate change predictions into a biodiversity impact assessment using species distribution models (SDMs)^21–24^. SDMs, fitted to current climate, land use, and other environmental variables (Supplementary Table 1) are extrapolated to conditions in 2070 under a range of climate and land use scenarios. Predictions of relative likelihood of occurrence are thresholded to examine changes in the ecologically suitable ranges for 1282 bird species in Vietnam and Australia^21–24^.

## Results

### Direct biophysical impacts dominate changing range sizes

**Figure 2:**
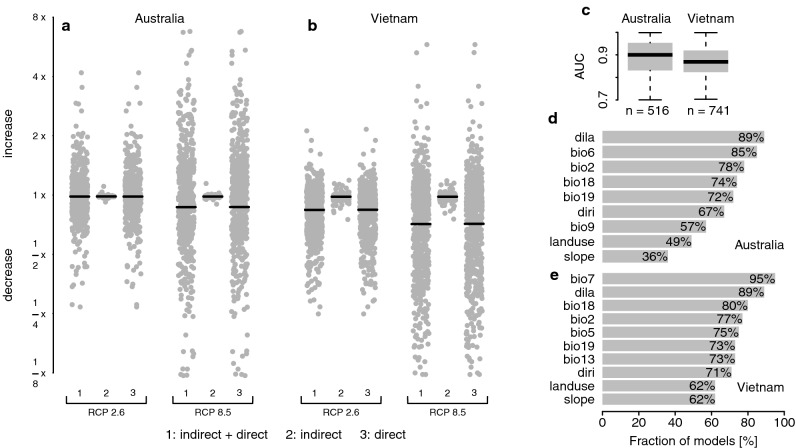
Predicted changes in species’ ecologically suitable ranges. (**a**, **b**) Illustration of multiplicative changes in species’ ecologically suitable ranges between present (2018) and 2070 for Australia and Vietnam respectively, under three treatments (1) “indirect + direct” (combined biophysical and socio-economic impacts of climate change), (2) “indirect” (net socio-economic impacts) and (3) “direct” (net biophysical impacts). Each point corresponds to a species, black bars are means of ecologically suitable range changes across all species. (**c**) A summary of cross-validated test Area Under the Receiver Operating Characteristics Curve values (AUCs)^25^ of models in the two regions as well as the respective number of models (*n*) retained (AUC > 0.7)^26^. AUC provides a measure of a model’s discriminatory performance in terms of how well test predictions discriminate between occupied and unoccupied locations^25,26^. (**d**,**e**) Fractions of models in which a predictor was used. Full names and definitions of all predictors can be found in Supplementary Table 1. Figure created in R version 3.5.1^27^ (https://www.R-project.org/).

For birds in both regions, we forecast major declines in ecologically suitable ranges, with severity of loss scaling with the severity of climate change (Fig. 2). Under RCP 8.5, a much higher number of species would be expected to experience decreases of more than half of their present ecologically suitable range compared with RCP 2.6, although variation in responses is also greater, indicated by the much wider spread of points (Fig. 2a,b). In Australia, mean suitable range decline under both pathways is not predicted to be as severe as in Vietnam and a smaller number of species is predicted to lose more than half of their suitable range. For both Vietnamese and Australian birds, predicting only under the indirect (land use change) effects of climate change results in little change to mean predicted outcomes for species (Fig. 2a,b), though some threatened species are predicted to lose significant suitable range within their current range due to indirect climate change impacts (see below). However, our analysis focuses on net climate change impacts on the economy; species ranges are likely to be affected more severely when also parametrizing other economic parameters to reflect future economic change. Mean predictions under combined direct and indirect effects do not differ to any notable degree from those made under direct biophysical effects only. Predictions under the first and third quartiles of bioclimatic variables across 15 Global Circulation Models (GCMs) show the same trends identified in the main results (Supplementary Figure 1).

SDMs for 1436 bird species were used in the analysis of the direct and indirect impacts of climate change on biodiversity. Discriminatory performance of the SDMs was assessed using cross-validated AUCs which varied between 0.7 and 1.0 with a mean of 0.90 in Vietnam and 0.87 in Australia (Fig. 2c), indicating very high discriminatory performance. We discarded models for 179 species with AUC < 0.7 (see “Methods” section). The predictor variables retained in the highest fraction of models were distance to lakes (*dist lakes*) in Australia and annual temperature range (*bio7*) in Vietnam. These are followed by *dist lakes* and precipitation of warmest quarter (*bio18)* in Vietnam, and by minimum temperature of the coldest week (*bio6*) and mean diurnal temperature range (*bio2*) in Australia. In Australia, *land use* was retained in about half the models. The very minor indirect (via *land use*) impact predictions arise because the changes in commodity demand predicted by the CGE model did not result in significant changes to land use in both regions (see below).

### Land use changes in response to climate change vary by region

**Figure 3:**
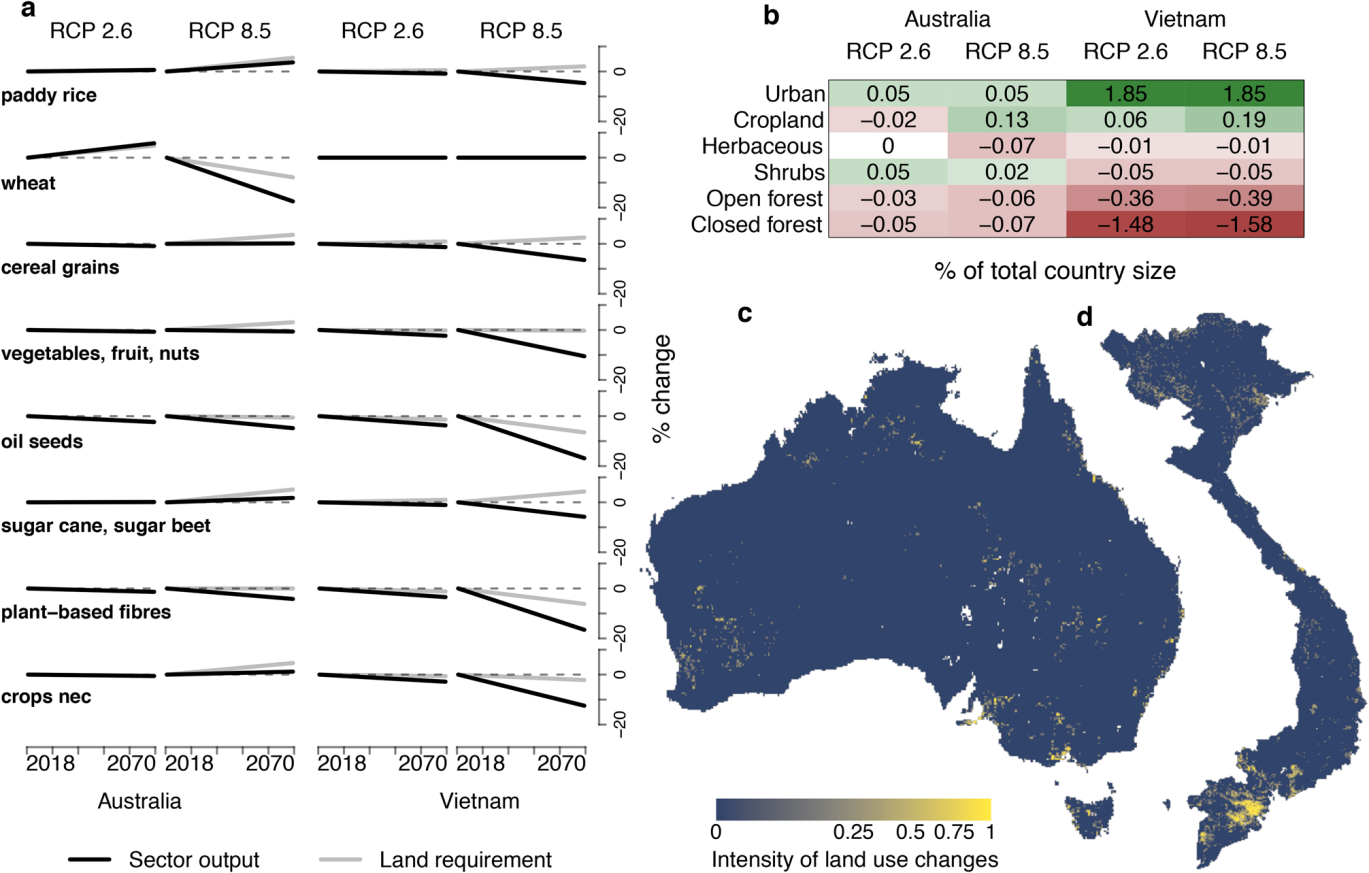
CGE and land use model results. (**a**) Future projections of commodity sector output and sector land endowments (the area required to produce output of a sector) from CGE model under RCP 2.6 and RCP 8.5. (**b**) Illustration of the percentage change of each land use in response to GTAP projections of crop sectors in (**a**) and FAO urban population projections, grouped by country and RCP, relative to the whole country size. (**c**,**d**), Intensity of predicted land use changes under indirect effects of RCP 8.5 in (**c**) Australia and (**d**) Vietnam. These maps are derived by aggregating predicted land use changes between any two classes under the indirect impacts of RCP 8.5 by factor 3. Figure created in R version 3.5.1^27^ (https://www.R-project.org/).

The total output of most agricultural crop sectors in both regions was predicted to decrease more with increasing climate change. In particular, in Vietnam, sectors such as oil seeds and plant-based fibres shrink by up to 20% under RCP 8.5 (Fig. 3a). The land requirements for each sector generally increase in proportion to the overall output of each sector. This is due to climate change impacts on crop yields as parametrised in the CGE-model: reductions in land productivity mean that more land is required to maintain sector outputs. Accordingly, in both countries, even while total outputs tend to decrease, land requirements of agricultural sectors remain approximately the same, or increase slightly (Fig. 3a).

**Figure 4:**
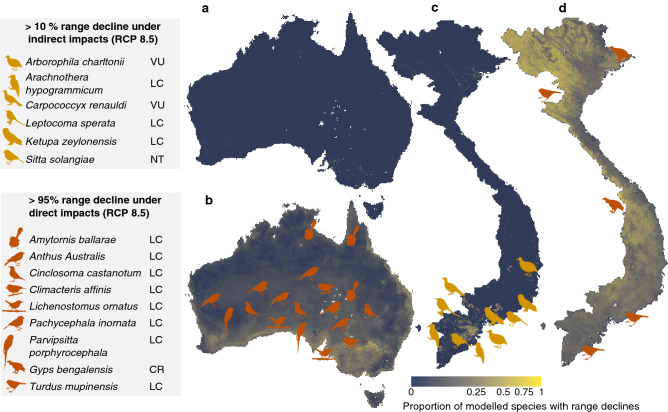
Mapping of range declines under RCP 8.5. (**a–d**), The proportion of avian species predicted to lose ecologically suitable range across Australia (**a**,**b**) and Vietnam (**c**,**d**) under the indirect (**a**,**c**) and direct (**b**,**d**) climate change impacts under RCP 8.5. Cell shading indicates the proportion of species predicted to lose suitable range in each cell. This identifies areas of declines in species’ suitable ranges from either indirect or direct impacts. The icons indicate locations of suitable range declines for severely affected species that lose more than 10% (indirect) and more than 95% (direct) of their suitable ranges overall. IUCN conservation status is given alongside taxonomic names (LC—least concern; VU—vulnerable; NT—near threatened; EN—endangered; CR—critically endangered)^29^. Icon credit—http://phylopic.org. Maps created in R version 3.5.1^27^ (https://www.R-project.org/).

The changes in land requirements for crop lead to an increase in cropland of < 0.5% of the total land area in both regions under RCP 8.5 and a very slight decrease in Australia under RCP 2.6 (Fig. 3b). Increases in urban land in both countries were modelled on FAOSTAT estimates of urban population growth^28^. In Australia, land use changes occur locally and are concentrated in coastal areas along the north-east, south and west of the continent, although some changes also occur further inland (Fig. 3c). In Vietnam, land use change is higher overall, with a particular concentration of change in the central-southern and northern coastal areas of the country, that also approximately coincide with the country’s major river deltas (Fig. 3d). Given that the distributions of most species are constrained, aggregated, and not random, small percentage changes in land use at the national scale still have significant impacts on some species locally (Fig. 4a,c). For example, species losing more than 10% of their currently suitable range under indirect impacts of RCP 8.5 in Vietnam include the vulnerable chestnut-necklaced partridge (*Arborophila charltonii*) and the near-threatened yellow-billed nuthatch (*Sitta solangiae*). These declines are highly localised and predominantly occur in the centre-south of the country (Fig. 4c). Direct climate change impacts are more severe: 324 and 362 species lose at least 10% of their suitable ranges under direct impacts of RCP 2.6 and RCP 8.5 respectively, with areas particularly affected across taxa under RCP 8.5 in the northern highlands and the central eastern parts of the country (Fig. 4d). Among the species losing more than 95% of their current suitable range under the direct impacts of RCP 8.5 are the Chinese thrush (*Turdus mupinensis)* and the critically endangered white-rumped vulture (*Gyps bengalensis*) (Fig. 4c,d).

In Australia, no species was found to lose more than 10% of its currently suitable range under indirect climate change impacts, although the black-throated whipbird (*Psophodes nigrogularis*) loses more than 5%. A higher number of species are affected by the direct impacts of climate change, with areas predicted to suffer particularly high suitable range declines along the southern and eastern coasts, the southwest and the southeast of the continent. In Australia, 188 and 230 species are expected to lose more than 10% of their suitable range under RCP 2.5 and RCP 8.5 respectively.

Amongst the Australian species losing more than 95% of their suitable range under the direct impacts of RCP 8.5 are the kalkadoon grasswren (*Amytornis ballarae*) and the Australasian pitpit (*Anthus Australis*) and a number of other species now categorised as of least concern (Fig. 4b). This highlights the potential dangers of climate change to species that we do not yet consider under threat, but for which extinction debts are accruing^30^.

The expected direct impacts of climate change impacts on many taxa are well researched and documented (i.e. increased extinction risks across taxa with accelerated climate change^31^, northward shifts of bird distributions in Great Britain under climate change^32^ and responses of bird abundance to climate change in the United States and Europe^33^). Our findings largely agree with these trends. In both Australia and Vietnam, climate change is likely to have extensive detrimental impacts on the climatically suitable ranges of birds. For many species, suitable ranges decline with increasing severity of climate change (Fig. 2) and under RCP 8.5, 24% of species analysed (in Vietnam) show likely declines in suitable ranges of greater than 50%, increasing their extinction risk in the country severely. Our analysis shows that subject to the assumptions of this work, the relative contribution of direct, biophysical impacts of climate change on biophysical suitability in our study area outweighs the contribution of indirect socio-economic impacts on habitat suitability via global commodity markets and resulting land use change, also taking into account the fact that climate change impacts on the suitability of land for particular uses. In Vietnam and Australia, bird species appear to be more severely impacted by the direct influence of changing climates than by its indirect impacts via commodity demand and land use.

## Discussion

Better understanding of climate change impacts on commodity demand and supply, and how those changes impact biodiversity should remain a research priority.

We predict economic change under two climate change scenarios, keeping all other aspects of the global economy at current baseline values. This way, we capture and isolate the effect of climate change on the economy. However, this approach omits other socio-economic processes that could affect supply and demand, such as population growth, changes to economic growth, energy efficiency, and shifts in social demands. These other factors may impact habitat and biodiversity through agricultural expansion, deforestation or urbanisation. While this study was designed to assess the net effects of direct and indirect climate change impacts on species as a first case study introducing our integrated assessment framework, these factors will be incorporated in future iterations that include an even more comprehensive CGE parametrization (i.e. full CGE baseline scenarios with socio-economic pathway narratives^34^ and integration of climate models in CGE analysis) and through improvements to current CGE methods by including, for example, stochastic effects of natural disasters in the CGE modelling.

Our results are valid for avian taxa in Australia and Vietnam under a number of assumptions about how commodity demand and supply, land use and biodiversity interact to deliver outcomes predicted by our integrated model. In our assessment framework, we follow a top-down modelling approach; within the architecture of our CGE model, climate change affects global demand and supply of many land-based commodities, requiring sector outputs as well as requirements of land to each sector to increase or decrease. However, mapped land use changes corresponding to changes in land endowments to different commodity sectors do not feed back into the CGE model. The inclusion of such feedbacks would increase the realism of both CGE and land use predictions, but detailed knowledge of local production systems and commodity markets are required to accurately parametrise such a model, and such models are computationally expensive^35^.

We chose not to produce SDMs for species with less than 20 occurrence records, to avoid the inflation of AUC for range-restricted species and species with very low prevalence^36^ and to assure sufficient discrimination between presences and background points^37,38^. Rare or spatially restricted species can be more vulnerable to localised impacts such as habitat loss^39^, but these effects are difficult to capture when biodiversity data are poor. We assumed unlimited dispersal ability in Vietnam and dispersal ability constrained to bioregions adjacent to those containing observation records in Australia. Disconnected patches of potential habitat outside of observed ranges (but within adjacent bioregions in Australia) were counted in future predictions, regardless of whether those areas were functionally linked (by suitable or traversable habitats) to the observed range and thus within the dispersal range of species. This may lead to an over-estimation of habitat utilisation and a commensurate underestimation of both direct and indirect impacts of climate change, particularly for taxa with a low dispersal ability that rely on small pockets of habitat within their range and are unable to reach disconnected patches of potential habitat. The importance of connectivity as a key component of habitat structure is well known^40^ and crucial for population viability in many species with low dispersal ability^41,42^. The parametrization of species’ dispersal ability and explicit modelling of landscape structure in response to land use change would allow for the inclusion of these fragmentation effects. This may be particularly important when our framework is extended to non-avian species.

While we found that total agricultural sector outputs decrease in both Vietnam and Australia, decreases in land productivity mean that land use in production for some agricultural commodities were predicted to increase slightly. We assumed a global economic equilibrium in which commodities can be substituted through trade between regions, thus implying that global demand for land-based commodities is serviced by regions that benefit from a comparative advantage under climate change. Where comparative advantage is due to increases in land productivity (land use efficiency), additional land may not be required to increase outputs. However, where this advantage is due to other economic mechanisms and not driven by the cost of converting additional land for production, more land may be allocated to agricultural or other commodity production, increasing habitat loss. For example, in Canada, our CGE model predicted an increase of wheat sector output by over 37% under RCP 8.5, while land endowments increase by only 14% due to increases in land use efficiency. In India, wheat output is estimated to increase by 8% under RCP 8.5, while land endowments to the wheat sector increase by 6%, suggesting much lower land use efficiency in India than in Canada (see Supplementary Figure 2 for a global, country-wise mapping of projected changes in sector outputs and land endowments of the wheat sector). Despite lower land use efficiency, wheat production in India still grows, because growth is economically feasible as long as it is not limited by factors arising from the sector’s context in domestic and international markets. In both countries, increases in land use lead to agricultural expansion, but in Canada more wheat can be produced per unit land and areas lost to wheat farming are likely to be much smaller per produced unit than in India. Nonetheless, if wheat production occurs in parts of Canada that were previously in, for example, natural prairie, then significant biodiversity losses may occur. Our framework provides in-depth insight into the links between sectors and regions and allows for a better understanding of global shifts in land requirements, enabling the fine-scale identification of hotspots for production, agricultural expansion and ultimately habitat destruction under consideration of the global economic processes.

In this first implementation of our framework we could capture and quantify principal relationships between climate change, the global economy, land use and avian habitat. Future uses of our approach could include regional and global biodiversity assessments following individual policy shocks, such as the introduction or abolishment of taxes or international trade deals, or could seek to capitalize on existing narratives of socio-economic futures and climate change pathways (so-called *Shared Socio-economic Pathways*)^34^ to parametrise climate adaptation policies, sustainable development goals and other aspects of socio-political transitions within the CGE modelling. Expanding consideration of biodiversity to include non-avian taxa and explicitly dealing with the role of connectivity and dispersal will enable a more comprehensive assessment of biodiversity impacts under socio-economic change. A key feature of our approach is that it provides opportunity to downscale country-level commodity demands to spatial explicit land use changes and biodiversity impacts, enabling a more meaningful analysis of the habitat and biodiversity implications of economic shocks or the implications of trade than have previously been possible.

Better integration of models and scenarios of biodiversity is required to guide evidence-based climate adaptation strategies and to chart progress toward sustainable development goals^43^. Our approach to integrating economic, land use and biodiversity values into a single model capable of high resolution, spatially-explicit predictions of land use and biodiversity outcomes provides information in a form that can be used directly by planners and managers. While spatial predictions of biodiversity and land use change have been available for decades, being able to place these predictions coherently in a global economic context is a new and exciting development that will bring a new level of relevance and realism to predictions in the eyes of policy and decision makers.

## Methods

### Study area

We focussed our analysis on Vietnam and Australia because the countries provide unique socio-economic contexts, while hosting a similar number of bird species that are vulnerable, endangered or critically endangered^44,45^. SDMs for Vietnam were built using data from a 30° × 30° tile that comprises large parts of Southeast Asia. This enabled us to capture the occurrence of bird species present in Vietnam on larger environmental gradients.

### Climate change

We chose Representative Concentration Pathways RCP2.6 and RCP8.5^46^ to include two extremes of the expected radiative forcing levels. For each pathway, we acquired the 2070 predictions of 19 bioclimatic variables^47^ from 15 GCM of Coupled Model Intercomparison Phase 5 (CMIP5)^48^. To capture variation between GCM predictions, we determined the cell-wise first, second and third quartiles each of the 19 variables across the 15 GCM (Supplementary Table 2).

Main results were derived by predicting land use and species distributions under the medians of these variables. We predicted both land use and species distributions under the first and third quartiles to approximate the range of outcomes for species across all 15 GCM (Supplementary Figure 1). In CGE models, we included the parametrization of both climate change pathways proposed by Roson & Satori^4^.

### CGE models

We developed an inter-temporal Global Trade and Analysis Project (GTAP) model^49^ to simulate changes in production under different climate change scenarios. CGE models use input–output-tables derived from national economic census data. These tables represent the inputs required in each economic sector to produce outputs and meet household and government demands (both nationally and internationally), which in turn are affected by prices and thus supply. Sectors are linked within each national economy, but also between economies. Producers in each country can produce various commodities to sell to domestic or foreign households and governments. Households and governments generate their income from selling (to producers) productive input factors (land, capital, labour, etc.) and through taxes. In our version of GTAP, the total land area (land endowment) from which allocations are made to crop sectors (land requirements) can be changed in the baseline. Therefore, land supply is not necessarily fixed, as is the case in most other GTAP models.

Estimations within GTAP are carried out relative to this baseline supply and we convert relative changes in agricultural land requirements to absolute changes in cropland by using their respective shares in the total harvested area for a by-sector-weighting of the average relative change of all classes (Supplementary Table 3). This weighted average change is applied on the current area under cropland to derive a future value. Accordingly, there is a direct proportional link between changes in land requirements and changes in the total area of agricultural land and the total area under agricultural land can change at the expense, or to the benefit, of other classes.

Since our GTAP model is a general equilibrium model, any shock to productivity will affect both the demand and supply of an agricultural commodity. The marginal cost of production generally increases (with the loss of productivity). At the same time, the income of households in the model will also be affected by the change in productivity as incomes are derived from selling (or renting) productive factors (land, capital, labour, and natural resources). As a result, prices change to equalize demand and supply and a substitution effect between agricultural commodities and between agricultural and other commodities takes place. As a result, demand does not stay constant. The contraction or expansion of the production of a particular crop is a result of interaction between demand and supply for that crop (both domestically and internationally).

Our inter-temporal GTAP model uses the GTAP 9 database^50^, which is subdivided into 139 regions and 57 commodity sectors^50^ and extends the GTAP model by replacing the recursive dynamic module of the current GTAP model with a forward-looking dynamic (or inter-temporal) module, where the producer can optimise profits overtime^51,52^. The inter-temporal GTAP model allows optimal investment behaviours, in which producers in each country can adjust their decisions based on the impacts from both past and foreseeable future events. Agents in the model can react to future threats long before their full realisation^52^. This makes the model a perfect tool for the simulation of future phenomena like climate change.

Following Roson & Satori^4^, climate change impacts in our GTAP model are realised as shocks to land supply and agricultural and labour productivity. The reduction in endowments of productive land and productivity negatively affect the production of commodities. Agricultural commodities are expected to be the most affected. With production shrinking more in some commodities than others, the price will adjust to balance the demand and supply of commodities. As a result, there is a substitution effect between domestically produced products and their competitive imports along with a substitution effect in factors of production (such as land), balancing demands between sectors.

Unlike the Kompas et al.^52^ approach, which relied on a one-step simulation approach, here we apply a multi-step simulation approach allowing the shocks to be applied into smaller successive intervals combined with extrapolation techniques to further enhance the simulation accuracy (see Horridge et al.^53^ and Pearson^54^ for details on multi-steps CGE solution methods). The solution of the inter-temporal GTAP model in this paper has been carried out within a parallel computing platform^19,55^ with the use of PETSC^56–58^ and HSL^59^ libraries.

### Land use models

We reclassified a global land use map to 8 land use classes (urban, cropland, herbaceous ground vegetation, shrubland, open canopy forest, closed canopy forest and wetlands and barren land) (Supplementary Table 1 for full list of data sources). Changes in urban land were estimated using estimates of urban population changes^60^ and adjusting the amount of land under this class, assuming that urban population density remains steady through time. Future applications of this work will establish links between land use classes related to forestry and livestock-raising, as has been demonstrated recently^17^.

We predicted land use maps under both pathways in 10-year time steps, using an R implementation (R package ‘lulcc’^61^) of the Conversion of Land Use and its Effects at Small regional extents (CLUE-S) model by Verburg et al.^62^. First, we determined the local suitability for different land uses through logistic regression of land use against the linear combination of a range of biophysical and socio-economic drivers in Generalised Linear Models (GLMs), from 15,000 randomly sampled pixels in each region (Supplementary Table 1 for a detailed list of data, Supplementary Figure 3 for effect sizes of predictors in each GLM). The selection of variables for land use suitability models was based on work by Verburg et al.^63^). Correlation analysis eliminated highly correlated predictor pairs (Spearmen’s rank correlation coefficient ≥ 0.7), always keeping the predictor whose highest correlation with any other remaining predictor was smaller, to maximise independent information retained in the final set. The final predictor sets were checked against literature^64,65^. We discarded a small number of predictors using cross-validated Lasso penalisation in the ‘glmnet’ R-package^66^ and used the reduced predictor sets to build GLM and predict to future timesteps by interpolating GCM-predicted WorldClim variables (Supplementary Table 2 for used GCM). GLM predictions produced maps of the landscape’s potential suitability for each land use class. Transitions between classes were constrained through a matrix detailing possible transitions (Supplementary Table 4). We specified conversion elasticities of each class (total possible turn-over within each class) based on literature^61,62^.

Projected demand changes were allocated iteratively until estimated land area demands were met^20^. Competition between land uses is handled in CLUE-S by allocating the land use with the highest predicted suitability in a given cell, accounting for conversion elasticity and allowed transitions. We masked category I and II protected areas^7^, precluding these areas from land use changes. Since there was no CGE-modelled future demand for herbaceous ground vegetation and shrubland, as well as the forest classes, the overall amount of area allocated to those land uses was what was not allocated to satisfy projected agricultural and urban demands. The proportional allocation between each of these residual classes was determined based on their mean predicted suitability in the landscape.

### Species distribution models

Correlative species distribution models (SDM) can predict responses of species to changing environmental conditions by extrapolating from the covariate space in which they were observed^21–24^. The MaxEnt software package^67^ (ver. 3.3.3 k) was used to fit SDMs for 656 bird species in Australia and 739 bird species in Vietnam, using presence-only data from the Global Biodiversity Information Facility (GBIF)^68^. We filtered records to retain observations from 1950 to 2018 with more than or equal to 20 occurrence points. We included a range of biophysical, topographic and socio-economic predictors as well as land use (Supplementary Table 1). Correlation analysis eliminated highly correlated predictors (see above). Literature review ensured that final predictor sets were ecologically meaningful to avian species across taxa^32,69–71^ at our aspired scale. We kept 9 predictors for Australia and 10 predictors for Vietnam, including 5 and 6 climate predictors respectively.

Sampling bias is a pervasive issue particularly affecting presence-only data that is often sampled opportunistically. We estimated sampling effort in response to demographic and topographic predictors^72^ (Supplementary Table 1). By selecting background points proportional to sampling effort, the its effect on the location of presence records is largely eliminated as a form of bias^73^.

Predictions were made using the estimated quartiles and medians of bioclimatic variables and the according land use maps that were also predicted under quartiles and medians. We controlled overfitting by dropping predictors with a permutation importance < 1%. Test AUC were estimated via fivefold cross-validation of each model and final models built on all available records. Species for which only uninformative models were fitted (AUC < 0.7) were excluded^26^.

We recorded the log ratio of the respective number of cells with relative likelihoods predicted above MaxEnt’s MaxSSS threshold^74^ (where the sum of model sensitivity and specificity is maximised) between the present (2018) and the future time step (2070) as a measure of change. In Australia, we constrained this change estimation for each species to bioregions containing records of the species, and adjacent bioregions^75^.

### Software and data

All data preparation and modelling for land use and SDMs was conducted in R (version 3.5.1)^27^, using packages ‘lulcc’^61^ for land use simulations and ‘dismo’^76^ for MaxEnt^67^ models. All analyses and spatial predictions of the land use model and SDM were performed at 0.5 arc-minute resolution; approximately 1 km at the equator. SDM building and predictions were computationally expensive and required up to 50 GB of working memory on 12 parallel cores.

## Supplementary Information

Supplementary Tables.

## Data Availability

Sources for data used in land use and species distribution modelling are listed in Supplementary Information. Direct download links to these data sets are available in the code repository accompanying this study (see below). We provide outputs of the CGE, land-use and species distribution models in a data repository (https://doi.org/10.26188/5ce25391e5b60). The GTAP database that underpins GGE modelling is available from GTAP under license. A link to a repository with the CGE modelling code that contains details of parameters settings for global economic models and detailed commodity demand output tables for each of the scenarios modelled is published with the code repository accompanying this study. All R-code for land use and species distribution modelling is available online (10.5281/zenodo.4461105).
