# Supplementary material for: Assessing biophysical and socio-economic impacts of climate change on regional avian biodiversity
Source: Sci Rep. 2021 Feb 8;11:3304. doi: 10.1038/s41598-021-82474-z (PMC7870812; doi:10.1038/s41598-021-82474-z)
Supplement: Supplementary file 1 — Supplementary Tables. [file 41598_2021_82474_MOESM1_ESM.pdf]

Supplementary Information for:

Assessing biophysical and socio-economic impacts of climate change on biodiversity

Authors: Simon Kapitza, Pham Van Ha, Tom Kompas, Nick Golding, Natasha C. R. Cadenhead, Payal Bal and Brendan A. Wintle

Corresponding author: Simon Kapitza; simon.kapitza.research@gmail.com

This PDF file includes:

Supplementary Tables 1–5

Supplementary Figures 1–3

References for Supplementary Information

Supplementary Table 1 | Climate, other biophysical, and socioeconomic predictors used as initial input to bias model, SDM, and land use model. Predictor choices were made based on literature. The initial predictor sets were reduced using correlation analysis.

Short name	Long name	Chosen?			Source
		**Bias**	**SDM**	**Land use**	
	**Climate predictors**				1
bio1	Annual mean temperature		x	x	
bio2	Mean diurnal range		x	x	
bio3	Isothermality		x	x	
bio4	Temperature seasonality		x	x	
bio5	Maximum temperature of warmest month		x	x	
bio6	Minimum temperature of coldest month		x	x	
bio7	Temperature annual range		x	x	
bio8	Mean temperature of wettest quarter		x	x	
bio9	Mean temperature of driest quarter		x	x	
bio10	Mean temperature of warmest quarter		x	x	
bio11	Mean temperature of coldest quarter		x	x	
bio12	Annual precipitation		x	x	
bio13	Precipitation of wettest week		x	x	
bio14	Precipitation of driest week		x	x	
bio15	Precipitation of driest month		x	x	
bio16	Precipitation of wettest quarter		x	x	
bio17	Precipitation of driest quarter		x	x	
bio18	Precipitation of warmest quarter		x	x	
bio19	Precipitation of coldest quarter		x	x	
bioregions	Bioregions in Australia		x		9
	**Other biophysical predictors**				
roughness	Roughness	x		x	3
slope	Slope		x	x	3
srtm	Elevation		x	x	3
diri	Distance to Rivers		x	x	4
dila	Distance to Lakes		x	x	4
dico	Distance to Coast			x	4
nitro	Soil Nitrogen Content			x	5
sawc	Soil Available Water Content			x	5
carb	Soil Carbon Density			x	5
bulk	Soil Bulk Density			x	5
	**Socio-economic predictors**				
pa	Protected Area	x		x	6
diro	Distance to Roads	x		x	7
dibu	Distance to Built-up Areas	x		x	8
popdi	Population density	x			9
landuse	Land use – Urban		x	x	10
	Land use – Cropland		x	x	
	Land use – Herbaceous vegetation		x	x	
	Land use – Shrubs		x	x	
	Land use – Open Forest		x	x	
	Land use – Closed Forest		x	x	
	Land use - Herbaceous wetlands, moss and lichen		x		
	Land use - Bare soil and sparse vegetation		x		

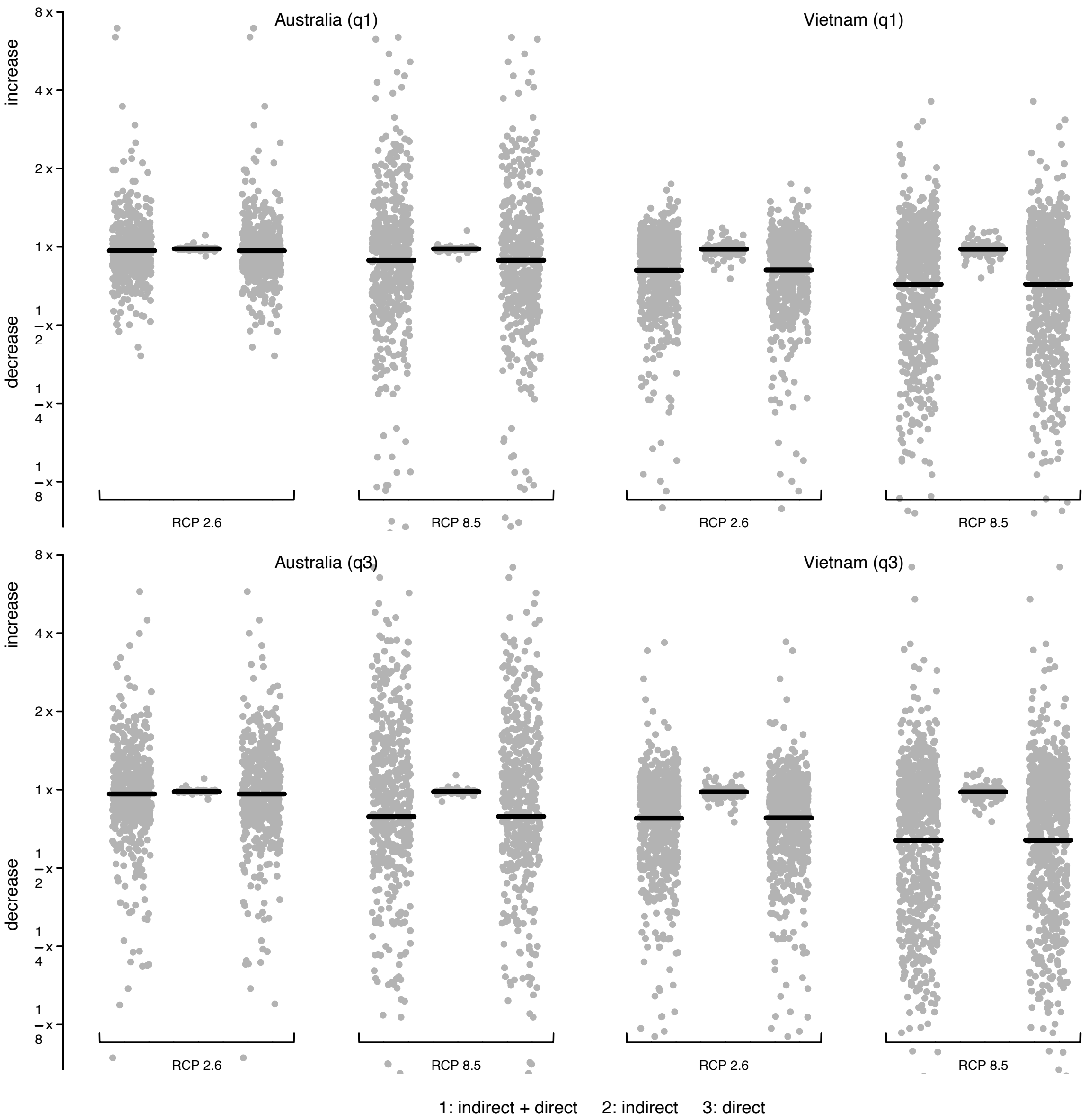

Supplementary Figure 1 | Predicted change in mean potential habitat in Australia (**a** and **c)** and Vietnam (**b** and **d)** under the first quartile of GCMs (**a** and **c)** and the third quartile of GCMs (**c** and **d**). Main results are predictions under the cell-level medians. Plots are constrained on the y-axis to < 8x and > 1/16 x for visual clarity. Figure created in R version 3.5.1[11] (https://www.R-project.org/).

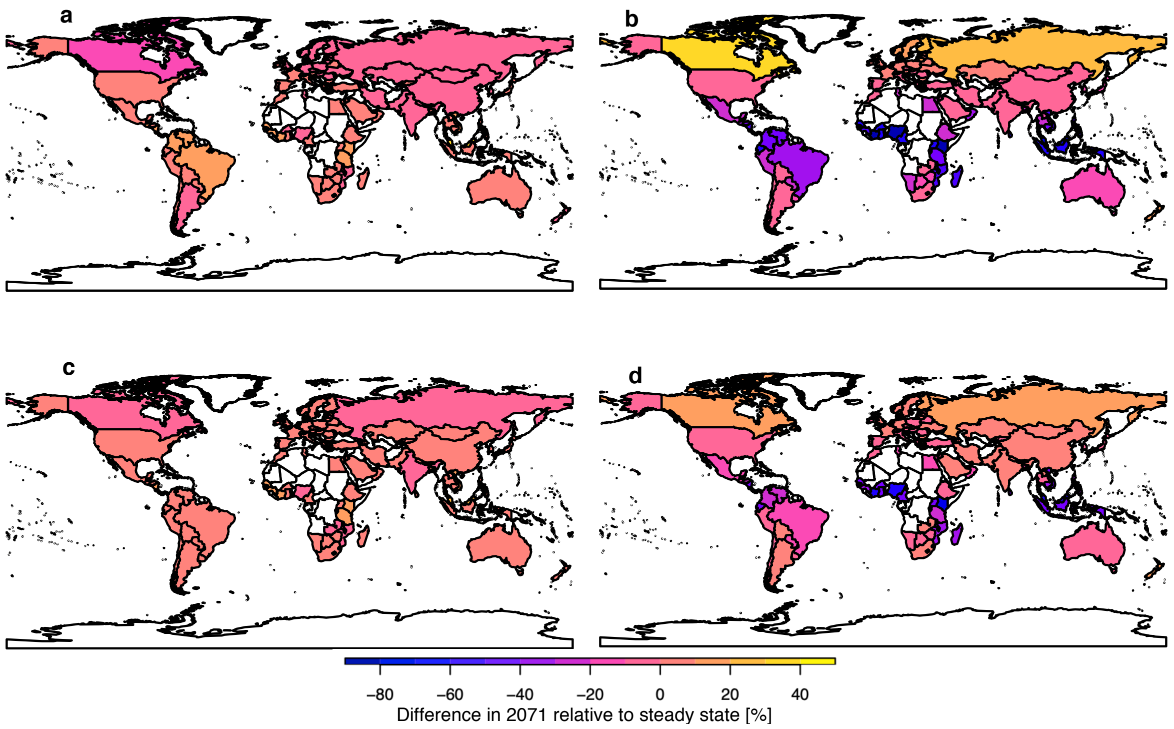

Supplementary Figure 2 | Relative changes in total wheat sector outputs (**a**, **b**) and land endowments (**c**, **d**) of GTAP 9 countries under RCP 2.6 (**a**, **c**) and RCP 8.5 (**b**, **d**). The % changes are relative to the economy after a forward propagation of the current economy without any scenario assumptions. Figure created in R version 3.5.1[11] (https://www.R-project.org/).

Supplementary Table 2 | List of Global Circulation Models (GCM). Downscaled outputs from these models were used to estimate cell-level medians and first and third quartiles of cell-level predictions of 19 biolcim variables. Data are available through WorldClim [12]

GCM	Source
BCC-CSM1-1	Beijing Climate Center, China Meteorological Administration
CCSM4	University of Miami - RSMAS
CNRM-CM5	Centre National de Recherches Météorologiques / Centre Européen de Recherche et Formation Avancée en Calcul Scientifique
GFDL-CM3	NOAA Geophysical Fluid Dynamics Laboratory
GFDL-ESM2G	NOAA Geophysical Fluid Dynamics Laboratory
GISS-E2-R	NASA Goddard Institute for Space Studies
HadGEM2-AO	Met Office Hadley Centre (additional HadGEM2-ES realizations contributed by Instituto Nacional de Pesquisas Espaciais)
HadGEM2-ES	Met Office Hadley Centre (additional HadGEM2-ES realizations contributed by Instituto Nacional de Pesquisas Espaciais)
IPSL-CM5A-LR	Institut Pierre-Simon Laplace
MIROC-ESM-CHEM	Japan Agency for Marine-Earth Science and Technology, Atmosphere and Ocean Research Institute (The University of Tokyo), and National Institute for Environmental Studies
MIROC-ESM	Japan Agency for Marine-Earth Science and Technology, Atmosphere and Ocean Research Institute (The University of Tokyo), and National Institute for Environmental Studies
MIROC5	Atmosphere and Ocean Research Institute (The University of Tokyo), National Institute for Environmental Studies, and Japan Agency for Marine-Earth Science and Technology
MPI-ESM-LR	Max-Planck-Institut für Meteorologie
MRI-CGCM3	Meteorological Research Institute
NorESM1-M	Norwegian Climate Centre

Supplementary Table 3 | Relative contributions of commodity sectors to the total area harvested in 2016 [13]. These values inform the weight of predicted land endowment changes when estimating total changes to crop land area.

Sector	Full name	Australia	Vietnam
c_b	sugar cane, sugar beet	0.020	0.018
gro	cereal grains	0.250	0.081
ocr	crops nec	0.094	0.129
osd	oil seeds	0.108	0.034
pdr	paddy rice	0.001	0.544
pfb	plant-based fibres	0.012	0.001
v_f	vegetables, fruit, nuts	0.019	0.193
wht	wheat	0.496	-

a

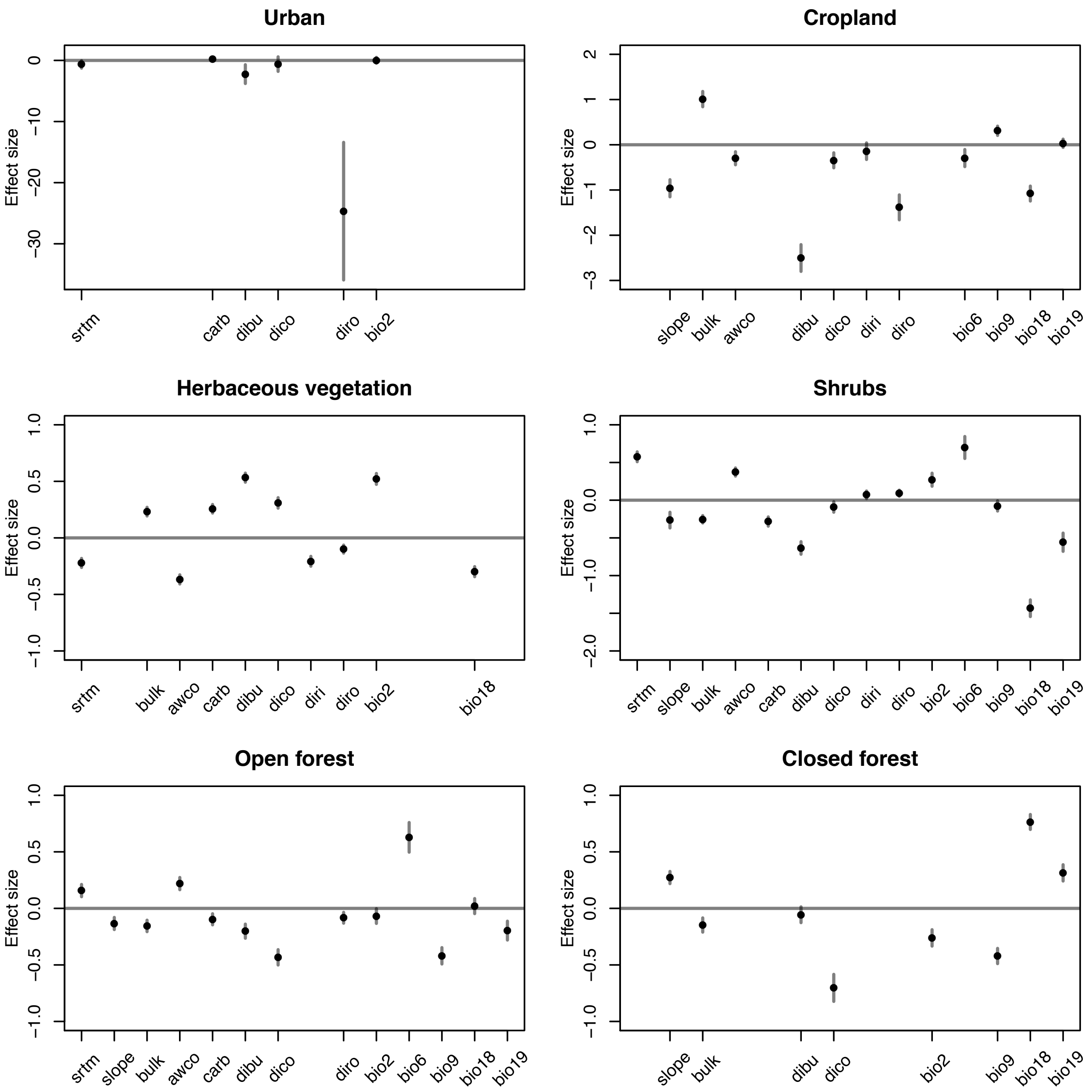
Urban
Effect size
0
−10
−20
−30
srtm
carb
dibu
dico
diro
bio2
Cropland
Effect size
2
1
0
−1
−2
−3
slope
bulk
awco
dibu
dico
diri
diro
bio6
bio9
bio18
bio19
Herbaceous vegetation
Effect size
1.0
0.5
0.0
−0.5
−1.0
srtm
bulk
awco
carb
dibu
dico
diri
diro
bio2
bio18
Shrubs
Effect size
1.0
0.0
−1.0
−2.0
srtm
slope
bulk
awco
carb
dibu
dico
diri
diro
bio2
bio6
bio9
bio18
bio19
Open forest
Effect size
1.0
0.5
0.0
−0.5
−1.0
srtm
slope
bulk
awco
carb
dibu
dico
diro
bio2
bio6
bio9
bio18
bio19
Closed forest
Effect size
1.0
0.5
0.0
−0.5
−1.0
slope
bulk
dibu
dico
bio2
bio9
bio18
bio19

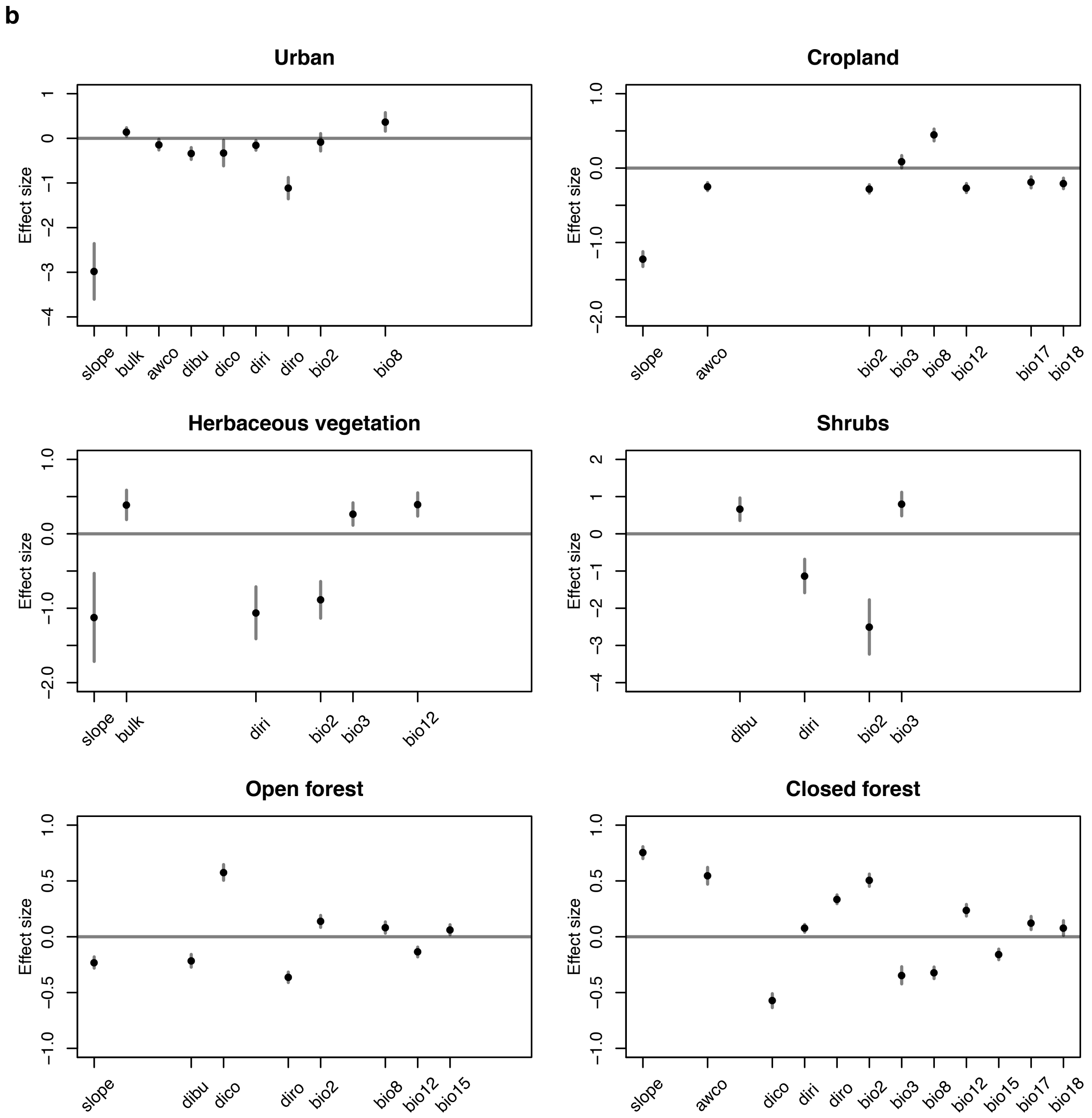

Supplementary Figure 3 | Effect sizes of predictors in land use suitability models. a, effect sizes of predictors in Australia and **b,** effect sizes of predictors in Vietnam. Predictors were standardised and we used cross-validated Lasso penalization for predictor selection. Error bars are indicated in grey. The sample size for model building was n = 20,000 in both countries. Figure created in R version 3.5.1[11] (https://www.R-project.org/).

Supplementary Table 4 | Transition matrix of land use model. 1 indicate possible transitions from the class of the row to the class of the column of the cell. 0 indicate when transitions are not possible.

**Class**	**Urban**	**Cropland**	**Herbaceous Ground Vegetation**	**Shrubs**	**Open Forest**	**Closed Forest**
**Urban**	1	0	0	0	0	0
**Cropland**	1	1	1	1	1	1
**Herbaceous Ground Vegetation**	1	1	1	1	1	1
**Shrubs**	1	1	1	1	1	1
**Open Forest**	1	1	1	1	1	1
**Closed Forest**	1	1	1	1	1	1

References

1. Hijmans, R. J., Cameron, S. E., Parra, J. L., Jones, P. G. & Jarvis, A. Very high resolution interpolated climate surfaces for global land areas. *International Journal of Climatology* **25**, 1965–1978 (2005).
2. Department of Sustainability, Environment, Water, Population and Communities. Interim Biogeographic Regionalisation for Australia (IBRA), Version 7 (Regions). Bioregional Assessment Source Dataset. http://data.bioregionalassessments.gov.au/dataset/70bb7ab7-e8a9-4be5-aa73-85bb22c2cb88.
3. NASA Land Processes Distributed Active Archive Center. Shuttle Radar Topography Mission (SRTM) 1 Arc-Second Global. in (2000).
4. Wessel, P. & Smith, W. H. F. A global, self-consistent, hierarchical, high-resolution shoreline database. *Journal of Geophysical Research: Solid Earth* **101**, 8741–8743 (1996).
5. Global Soil Data Task Group. Global Gridded Surfaces of Selected Soil Characteristics (IGBP-DIS). (2000) doi:10.3334/ornldaac/569.
6. IUCN and UNEP-WCMC. *The World Database on Protected Areas (WDPA)*. https://www.protectedplanet.net (2014).
7. Center for International Earth Science Information Network - CIESIN - Columbia. Global Roads Open Access Data Set, Version 1 (gROADSv1). (2013).
8. FAO (Food and Agricultural Organisation. Built-up Areas of the World (Vmap0). **First edit**, (1997).

9. Center for International Earth Science Information Network (CIESIN) Columbia University, International Food Policy Research Institute (IFPRI), The World Bank & Centro Internacional de Agricultura Tropical (CIAT). Global Rural-Urban Mapping Project, Version 1 (GRUMPv1): Population Density Grid. (2011).
10. European Union. *Copernicus Land Monitoring Service*. (2019).
11. R Development Core Team. *R: A language and environment for statistical computing*. (Foundation for Statistical Computing, 2008).
12. Hijmans, R., Cameron, S., Parra, J., Jones, P. & Jarvis, A. WORLDCLIM - a set of global climate layers (climate grids), version 1.4.
13. Food and Agriculture Organization of the United Nations (FAO). *FAOSTAT Statistics Database*. (2017).
